# Attenuation properties of hybrid nanocomposite film containing Ce_2_O, GO, and α-Al_2_O_3_ nanoparticles for high energy radiations

**DOI:** 10.1038/s41598-023-43212-9

**Published:** 2023-09-23

**Authors:** Mehdi Mahmoudian, Mahsa Radmehr, Khadijeh Alimoradlou, Asghar Zamani, Peyman Gozali Balkanloo

**Affiliations:** 1 Nanotechnology Department, Faculty of Science, Urmia University, Urmia, Iran; 2 Department of Organic Chemistry, Faculty of Chemistry, Urmia University, Urmia, Iran

**Keywords:** Energy, Chemistry

## Abstract

The use of diagnostic radiation in medical centers has spread due to the incidence of various diseases. Thus, it is essential that patients and medical staff wear protective clothing to protect themselves from their harmful effects. In the past, lead protective clothing has been used; however, the toxicity and heaviness of lead have limited the tendency to use these clothing. Recently, nanocomposites containing heavy element nanoparticles have been introduced as an alternative to lead coatings. In this study, hybrid nanocomposites containing ceria (CeO_2_), alumina (Al_2_O_3_), and graphene oxide (GO) nanoparticles were studied for this purpose. Ceria, alumina, and graphene oxide nanoparticles were mixed with polyethylenevinylacetate (EVA) dissolved in chloroform and casted on a glass plate to form nanocomposite films. The prepared nanoparticles and films were characterized by Fourier Transform Infrared Spectroscopy, Field Emission Scanning Electron Microscope, Thermal Gravimetric Analysis, and Energy Dispersive X-ray Analysis, and then the attenuation properties of the films against high-energy radiation (120 kV) were studied in two narrow and broad beam geometries. The results showed that hybrid films, despite having a lower percentage of nanoparticles, showed better attenuation properties, which indicated the synergistic effect of nanoparticles with different mechanisms in attenuating the radiations. The attenuation ability of these films was considerable due to their lower density compared to lead. The fabricated hybrid nanocomposite films with a suitable performance in attenuation of high-energy radiations used in therapeutic diagnostics, can be proposed as a suitable alternative to conventional lead clothing.

## Introduction

High-energy radiations such as X or γ-rays are widely used in hospitals and health centers for disease diagnostic^1^. On the contrary of beneficial advantages of these radiations in medical applications, different casual and specific effects on the human body are known^2,3^. Therefore, the patients and the technicians exposed to such radiations must be protected^4^.

Decreasing the intensity of ionizing radiation is one of the methods used for protection against high energy radiations known as attenuation^5,6^. This process can be occurred as a result of absorption or dispersion of radiation through the materials existing in the environment and is directly related to the atomic number of absorbents and also radiation energy^7,8^. Another important factor affecting this phenomenon is the attenuation coefficient, which is a quantitative parameter for the designation of attenuation system by the absorbent thickness. Hence, the type of the radiation and the possible interactions of the radiation and the material used as absorbent should be investigated in order to design and produce effective coatings for radiation protection^9,10^.

Different mechanisms are known to attenuate high-energy radiations. K edge absorption is one of the critical factors for attenuation investigation^11^. According to this mechanism, when the energy of a photon is greater than the electron’s binding energy at layer K, the energy can be completely absorbed; consequently, an electron is released from this layer leading to an abrupt enhancement of attenuation. This type of energy absorption is called “photoelectric absorption”^12,13^. The second mechanism of attenuation is caused by the partially energy loss of an X-ray photon during the interaction with substance directing to an increase in wavelength and can be named the Compton scattering phenomenon^14^.

In the past years, protection against radiation exposures during the diagnosing process for patients, technicians, physicians, and radiologists was provided by wearing some heavy metal or composite clothes^15,16^. Lead as an element with a high atomic number and density is one of the most used heavy metals for X-ray radiation attenuation^17^. However, despite the excellent characteristics of lead, its high weight, toxicity, and possible interactions with high energy radiations, which creates secondary radiation, requires further protection, and causes extra costs^18^. As a consequence, recently enormous efforts have been carried out to produce effective, lightweight and flexible protective clothes. Polymer composites have been used widely as an effective alternative in which lead is used as filler within the polymeric matrix. Moreover, some other elements such as Barium, Antimony, and Tungsten are used as fillers^19,20^.

The development of nanotechnology has introduced new ways in designing and manufacturing efficient protective clothes. Studies have shown that using some nanoparticles as fillers in the matrix of composites leads to highly effective protective coatings against X-ray irradiation^21^. Investigations have shown that nanoparticles used in polymeric nanocomposites have a more attenuating effect due to better dispersion in the matrix and greater interaction with radiation^22^. The effect of nanoparticle size on the attenuation of high-energy radiation has been investigated in several articles^23,24^. Botelho et al. showed that CuO nanoparticles embedded in the wax enhanced attenuation characteristics at low energies^25^. Nambiar et al. used Bismuth oxide nanoparticles in polydimethylsiloxane matrix in order to increase the protection characteristics of the coating^7^. Leatherday et al. designed some green lightweight lead-free Gd_2_O_3_/epoxy nanocomposite for X-ray protective coatings^26^. Magnetic nanocomposite films were fabricated for radiation shielding using Fe_3_O_4_ nanoparticles^27^. Mondal et al. designed flexible nano-structured nanocomposites with improved shielding properties using carbon nanofibers^28^.

As mentioned above, various types of nanomaterials and nanoparticles are used in protective coatings such as CeO_2_, GO, and Al_2_O_3_. CeO_2_ nanoparticles are effective for radio-protective coatings due to high atomic weight of Cerium, their antioxidant properties, and stability^29,30^, GO because of its planar structure^31,32^, and Al_2_O_3_ because of some unique properties^33^. In the present study, these nanoparticles were incorporated into a polyethylenevinylacetate (EVA) matrix, and used as a hybrid nanocomposite system to study the protective ability of the coatings in attenuation of the high energy radiation (120 kV). EVA was selected due to its flexibility, high nanoparticle loading capability and non-toxicity. Based on the performed literature review, in a few studies, the synergistic effect of nanoparticles in hybrid polymer nanocomposites for attenuation of high-energy radiations has been investigated. FTIR, FESEM, TGA, and EDAX techniques were used to characterize the nanoparticles and prepared coatings as well.

## Experiment

### Materials

Ethylene vinyl acetate with 28% vinyl acetate was provided from Hyundai Petrochemical of Korea. Chloroform was used as a solvent and purchased form Sigma Aldrich. α-Al_2_O_3_ (α-Alumina) nanoparticles bought from US Research Nanomaterials, with the size of 100 nm were used. A pure lead film with a purity of 99.968 and a thickness of 0.25 mm was purchased from Merck.

### Synthesis of CeO_2_ and GO nanoparticles

CeO_2_ nanoparticles were fabricated in the presence of walnut shell and cerium (III) nitrate hexahydrate, according to our previous study^34^.

GO was synthesized by the modified Hummers method in the previous study and used exactly the same way^35–37^.

### Fabrication of neat and hybrid nanocomposite films

To fabricate neat film, EVA (5 g) was added into chloroform (25 ml) in a round bottom flask, and was stirred with a magnetic stirrer to obtain a completely homogeneous solution at room temperature. This solution was spread on a clean glass plate with a diameter of 15 cm, maintained in a vacuum desiccator for 30 min to eliminate air bubbles and finally dried at 30 °C in an oven. The flexible film was separated and stored for tests.

Nanocomposite films were prepared similarly, except that the nanoparticles used were first dispersed completely by an ultrasonic homogenized (Hielscher UP200St) in chloroform (25 ml) and then EVA was added to the mixture. The thickness of the prepared films was about 2 mm. Different compositions were applied to prepare hybrid nanocomposite films, which can be seen in Table 1.

**Table 1:** Composition of the neat and hybrid nanocomposite films.

Samples	Additives		
	GO (wt%)	Nanoceria (wt%)	α-alumina (wt%)
M_1_	0	0	0
M_2_	4	0	0
M_3_	5	0	0
M_4_	7	0	0
M_5_	0	10	0
M_6_	0	25	0
M_7_	0	35	0
M_8_	0	0	25
M_9_	0	0	35
M_10_	0	0	50
M_11_	0	10	25
M_12_	4	10	0
M_13_	4	0	25
M_14_	4	10	25

### Characterization

FESEM, (Czech company TSCAN), was used to study the dimensions and morphology of the prepared nanoparticles and films. Sample treatment for taking cross-sectional images involved freezing the films in liquid nitrogen and breaking them. The samples were coated with a thin layer of gold before imaging.

The chemical structure of nanoparticles and films was investigated using Attenuated Total Reflectance -Fourier Transform Infrared Spectroscopy (ATR-FTIR), PerkinElmer Spectrum Version 10.03.02, in the range of 400–4000 cm^−1^.

The effect of nanoparticles on the thermal stability of films was studied by TGA, Linseis TP 1000. The heating rate was 10 °C/min, and the tests were performed under atmosphere conditions.

The attenuating performance of the fabricated films in high energy beams (X-rays) was evaluated using the dosimetry method. The dosimeter used was equipped with an ionization chamber (Piranha Red (RTI Electronics AB, Sweden)) and was connected to a computer via Bluetooth. The analyses were processed using Ocean software.

The used radiation source included a diagnostic X-ray device (Shimadzu X-ray device model 1/2P13DK Japan) with a focal point of 1.2 mm. The applied potential for the tests was 120 kV and the current and exposure time of the sample films were 200 mA and 100 ms, respectively. Test conditions were established in accordance with the standard American society for testing and materials (ASTM) F2547-06. Each test was repeated three times for the samples, and the obtained averages were reported as the results.

### Attenuation measurements

Dose values of primary (I_0_) and secondary radiations (I) were recorded using a Piranha dosimeter and used to calculate the attenuation percentage according to the following equation.

$$Attenuation=\left(1-\frac{I}{{I}_{0}}\right)\%$$

The attenuation values obtained for the films were compared with the attenuation ability of the pure lead layer.

**Figure 1:**
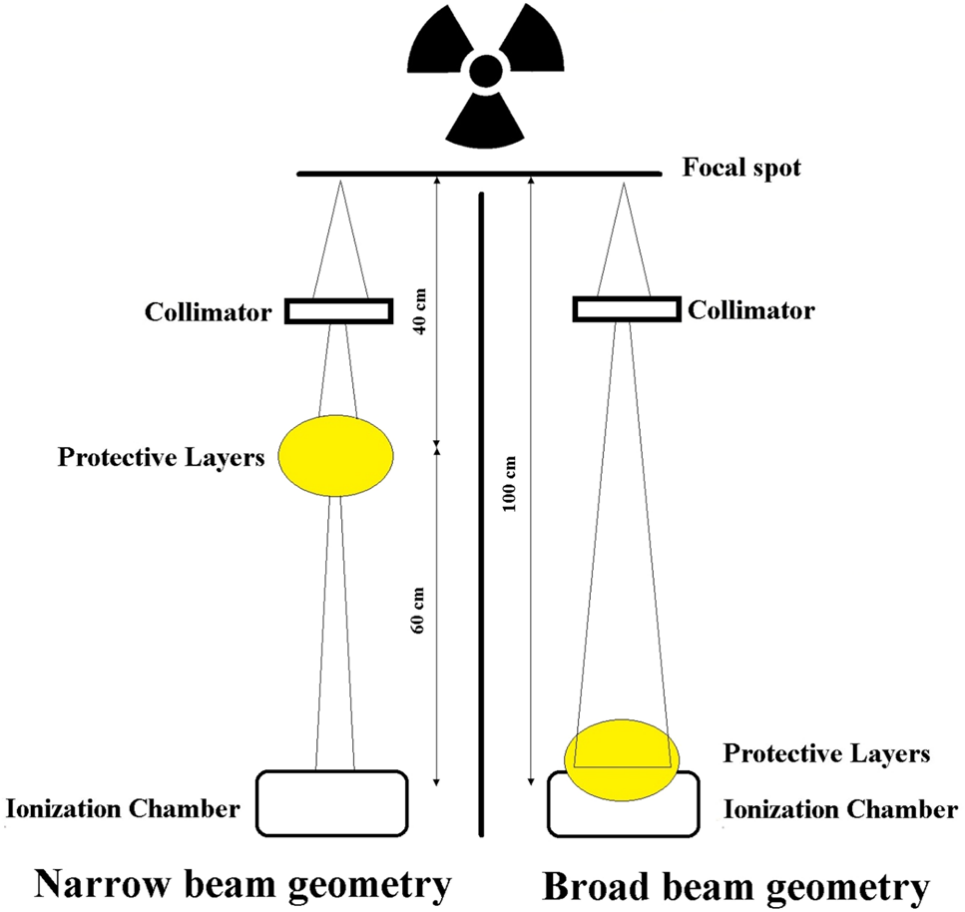
Configuration of narrow and broad beam geometries.

Dosimetric studies were performed under two configurations, which include narrow beam geometry, and broad beam geometry. The arrangement of the components in these two geometries is shown in Fig. 1.

In narrow beam geometry, the calculated attenuation belongs to the primary beams and the secondary photons produced are not detected. Beer-Lambert equation was used to determine the dose rate. In this equation, I_0_, I, and x symbols indicate the intensity of the incident and passing radiations, and the thickness of the target film, respectively and µ shows the linear attenuation coefficient.

$$I={I}_{0}{e}^{-\mu x}$$

In this equation, substituting the thickness multiplication in density instead of the usual thickness gives the attenuation calculated in terms of mass thickness.

In broad beam geometry, measurements were performed similarly, except that the target film samples were placed on the detector. Therefore, in this configuration, the scattered secondary photons and the fluorescence radiations obtained from the interaction of the primary radiations with the sample were also detected. As a consequence, the attenuation calculated in this configuration provides a more realistic measure of the film’s capability.

## Results and discussion

### Characterization of nanoparticles and fabricated nanocomposite films

In this study, three types of nanoparticles (ceria nanoparticles, GO, and α-Alumina) have been used to make hybrid nanocomposite films, which were identified using the FTIR and FESEM analyzes.

**Figure 2:**
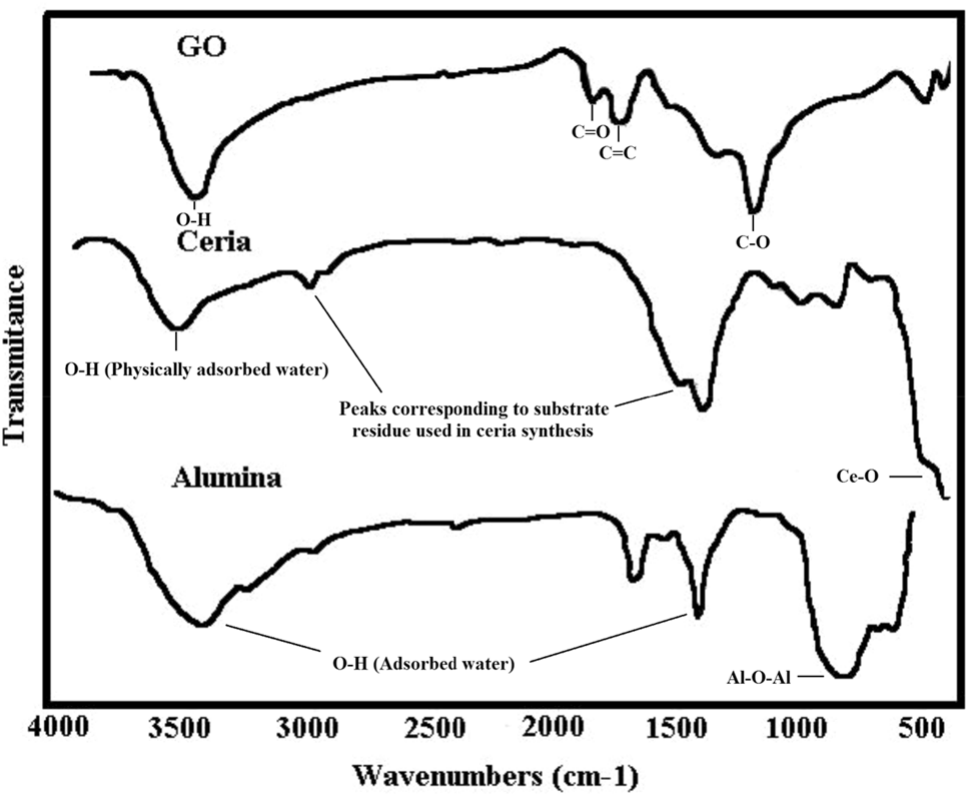
FTIR spectra of GO, ceria, and α-alumina.

The FTIR spectra are shown in Fig. 2. Characteristic peaks of GO can be seen at the 3400, 1720, 1650, and 1032 cm^−1^ wavelengths, which belong to the stretching vibrations of the O–H, C=O, C=C, and C–O bonds, respectively, and prove the successful synthesis of GO from graphite^35^.

The absorption peak observed at 3500 cm^−1^ in the spectrum related to ceria was attributed to the stretching vibrations of the O–H bonds in the physically adsorbed water. The sharp peak at 447 cm^−1^ was related to the stretching vibrations of the Ce–O bonds. The rest of the peaks (2950 and 1430 cm^−1^) in the spectrum may be due to residual impurities in the nanoparticles that result from the walnut template used to synthesize nanoparticles^34^.

α-alumina as a mineral compound has shown a peak at 819 cm^−1^, which was related to the stretching vibration of Al–O–Al bonds. Peaks appearing in 1642 and 3450 cm^−1^ were attributed to the stretching vibrations of water adsorbed on α-alumina and surface-attached hydroxyl groups^38^.

**Figure 3:**
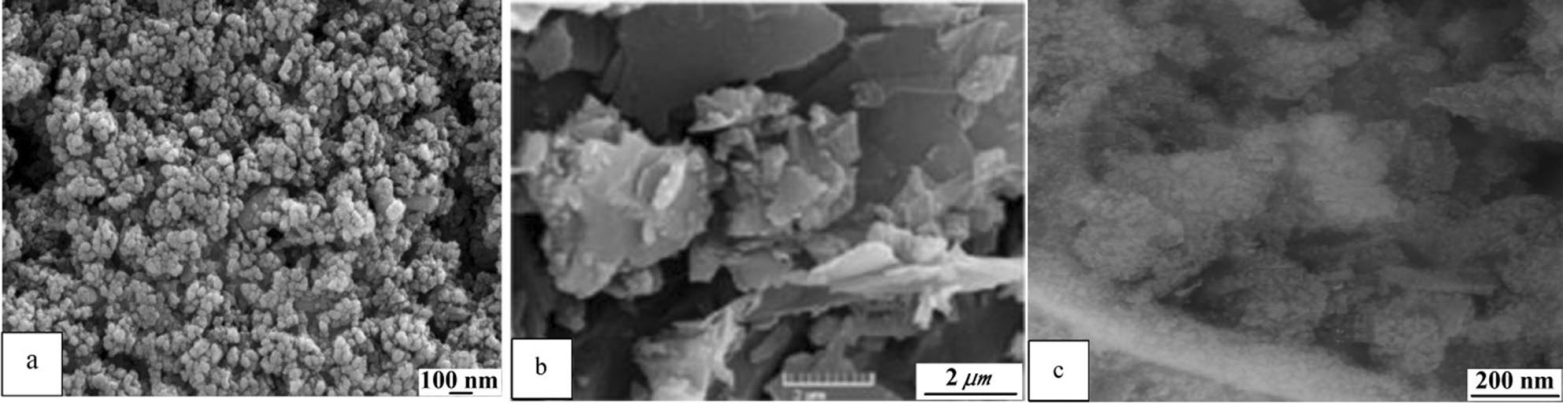
FESEM images of (**a**) α-alumina, (**b**) GO, and (**c**) ceria.

FESEM images of applied nanoparticles are represented in Fig. 3. As can be seen, α-alumina had a spherical shape with an approximate diameter of 75 nm and has an excellent dimensional uniformity (α-alumina size distribution is represented in Fig S2). GO plates were also well recognizable in the image, and the nanometer thickness of the layers was provable. It seemed that surface oxidation modification caused the destruction of layers, and the plates did not have the same surface area. Ceria spherical nanoparticles with a diameter of approximately 10 nm can also be seen in the relevant images (ceria size distribution can be seen in Fig S1). Remains of the walnut template in the image were seen. Accumulation of nanoparticles can also be observable in the image.

**Figure 4:**
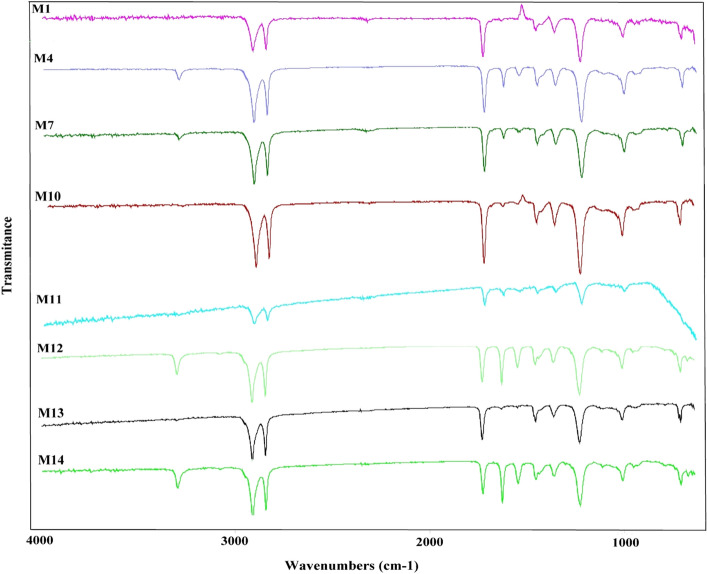
FTIR spectra of the neat and Hybrid nanocomposite films.

The prepared nanocomposite films were characterized by ATR-FTIR analysis to investigate possible changes in the chemical structure of the surface of films. The resulted spectra are shown in Fig. 4. The EVA neat film (M_1_, without any nanoparticles) showed the characteristic peaks at 2920, 2810, 1735, and 1245 cm^−1^, which were related to stretching vibration of C–H, C=O, and C–O bond, respectively. M_4_ film that contained 7 wt% GO had two additional peaks at 1640 and 3340 cm^−1^, which were attributed to the C=C and the hydroxyl groups, respectively. The next film M_7_, contained 35 wt% ceria showed a similar spectrum, so that the intensity of peaks at 1640 and 3340 cm^−1^ has decreased. M_10_, as the film containing 50 wt% α-alumina, showed a spectrum quite similar to pure film. The rest of the films were hybrid nanocomposites containing two (M_11_, M_12_, M_13_) or three (M_14_) types of nanoparticles and obtained almost similar spectra for them.

**Figure 5:**
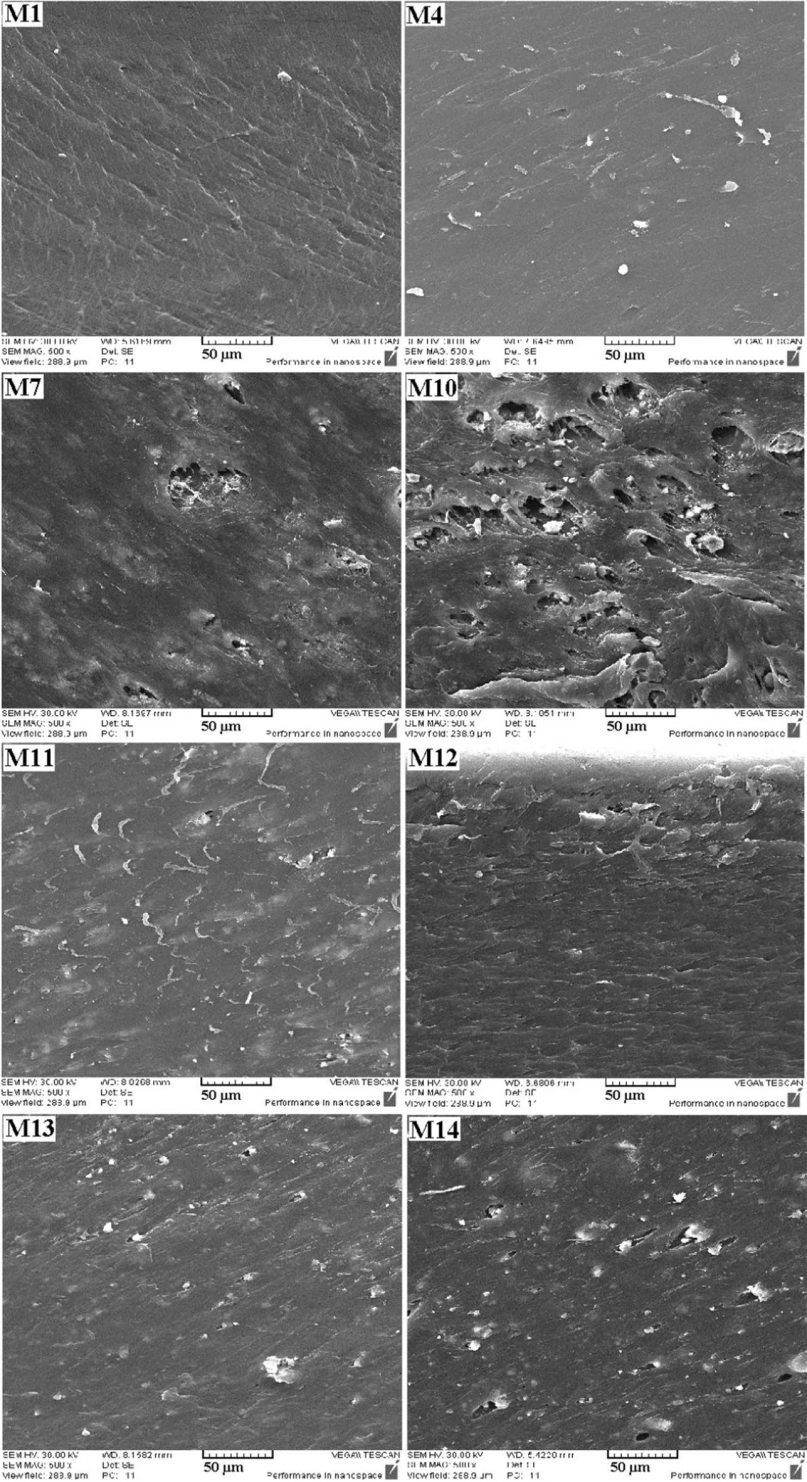
FESEM cross-sectional images of neat and hybrid nanocomposite films.

To study the morphology of the prepared films and the distribution of nanoparticles in the films, FESEM cross-sectional images of the samples were prepared. The results are shown in Fig. 5. The cross-section of the M_1_ film had a completely smooth surface and no external factors could be seen. However, in M_4_, M_7_, and M_10_ films, objects or defects in the cross-section could be detected. There were fewer defects in film M_4_, while the defects seen in films M_7_ and M_10_ were very significant, which was attributed to the high percentage of additives. In hybrid films (M_11_–M_14_), lower percentages of nanoparticles were used. The presence of nanoparticles in the cross-sectional images was clearly visible, while no significant defects were seen in the images.

**Figure 6:**
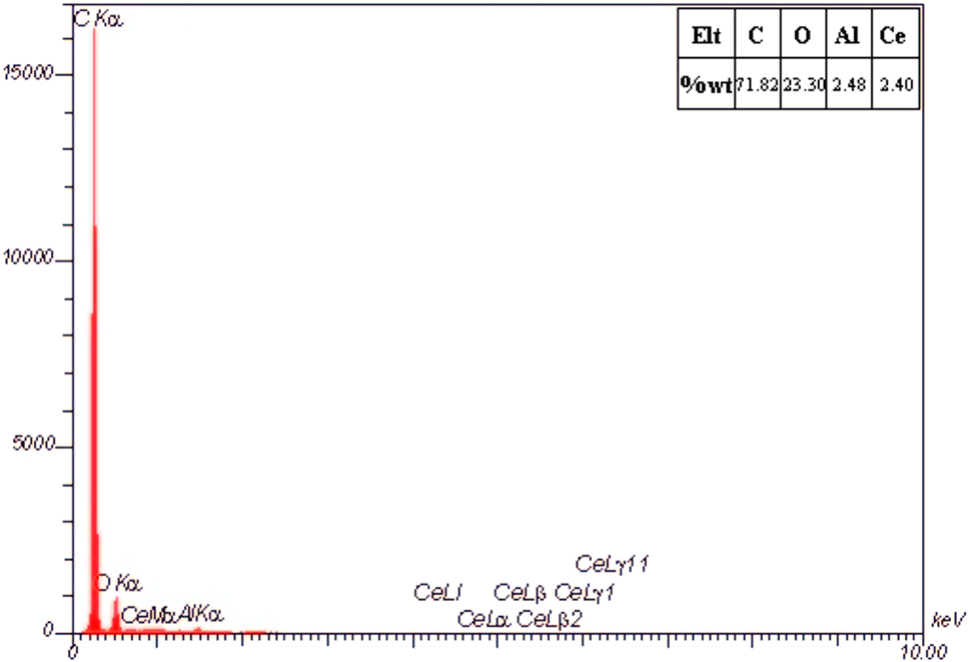
EDAX analysis of M_14_ film.

EDAX elemental analysis was performed on the surface of the hybrid nanocomposite film M_14_ to further confirm the presence of nanoparticles. The result is shown in Fig. 6. As can be seen, the presence of C, O, Al, and Ce elements on the surface has been proven. However, the percentage of elements did not match the amounts of used nanoparticles. This mismatch may be due to the high density of α-alumina and ceria nanoparticles, which cause them to precipitate during the drying process of the films.

**Figure 7:**
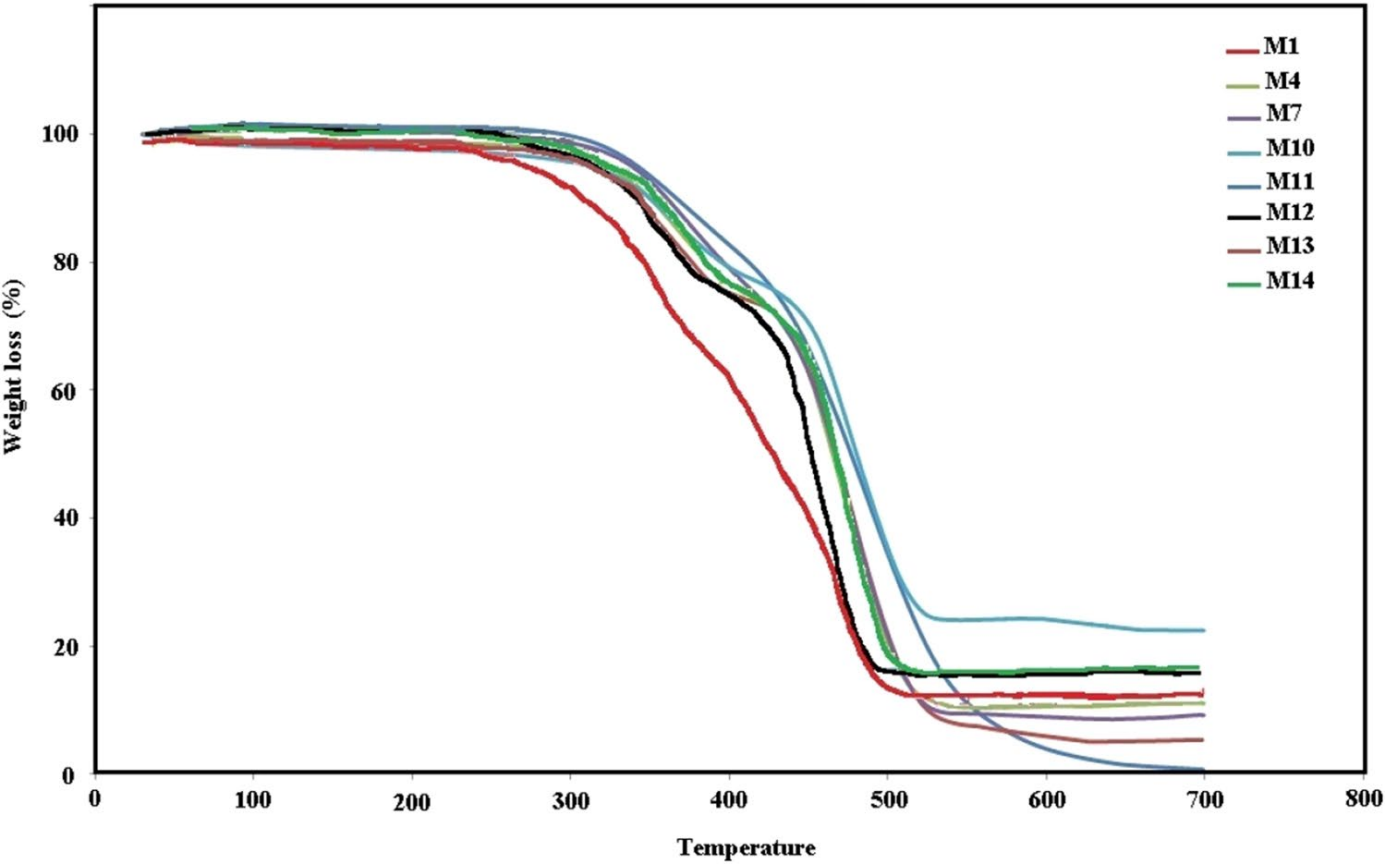
TGA thermograms of neat and hybrid EVA nanocomposite films.

The thermal stability of the prepared films was investigated by TGA analysis to survey the effect of nanoparticles. The resulting thermograms are shown in Fig. 7. Pure and hybrid nanocomposite films were degraded in two stages. In film M_1_ (the film without any nanoparticles), the first destruction has started at 250 °C, and the second stage of degradation occurred at 350 °C. In nanocomposite films, degradation temperatures shifted from 20 to 50 °C to higher temperatures and showed the effect of nanoparticles on the thermal stability of nanocomposites. The lowest thermal stability was seen in film M_1_, and the highest thermal stability belonged to film M_10_ (the film with 50 wt% alumina).

### Investigation of attenuation performance of the fabricated films

**Figure 8:**
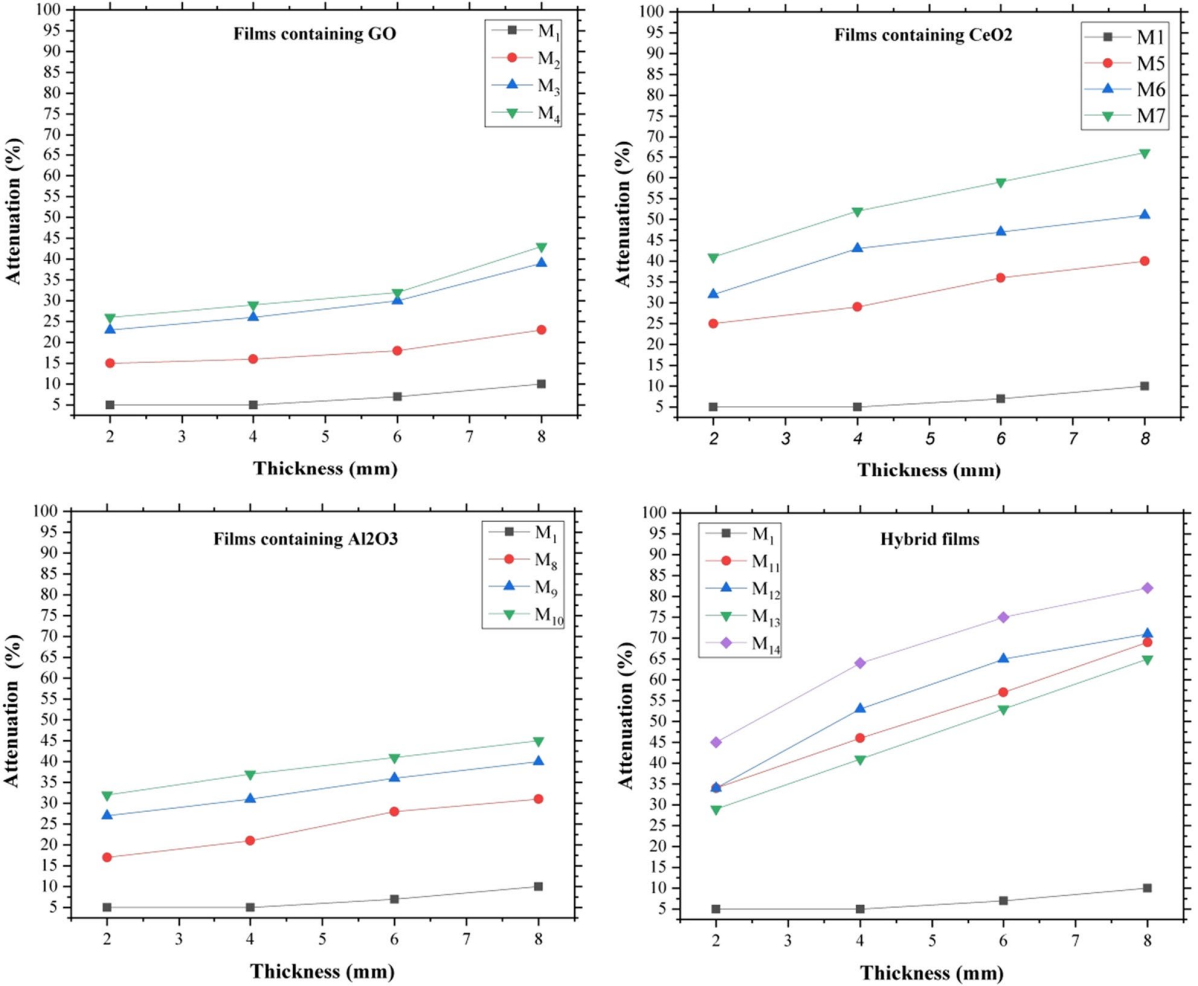
Attenuation of neat and hybrid nanocomposite films in terms of layer thickness in the narrow beam geometry.

Based on the performed leterature review, in this study nanoceria, alumina, and GO nanostructures were used as additives in the EVA matrix. These nanostructures were selected with nano dimensions due to their high surface area and different morphologies and proper interaction with incident radiations so that can be effective in attenuating high-energy rays. The attenuation performance of the films was investigated in two narrow and broad geometries. For this purpose, different thicknesses of the films presented in Table 1 were prepared, and radiations with 120 kVp energy were selected for irradiate the films. The results of the study of the effect of thickness on the attenuation of radiations in narrow beam geometry are shown in Fig. 8. Attenuation of pure EVA film was compared separately with films containing GO (M_2_–M_4_), ceria (M_5_–M_7_), alumina (M_8_–M_10_), and hybrid films (M_11_–M_14_). It was observed that the EVA film (M_1_) had the least attenuation, and the addition of nanoparticles increased the attenuation ability of films. The attenuation mechanism of nanoparticles is different. Attenuation of radiations by ceria and α-alumina nanoparticles occurs mostly through photoelectric adsorption, while the attenuation mechanism in the presence of GO nanoparticles is through the Compton phenomenon. This phenomenon is the predominant interaction in the collision of X-rays with elements with low atomic numbers and leads to the scattering of secondary radiation^39^. Ceria nanoparticles were more effective than other nanoparticles, and this was probably due to the predominance of photoelectric absorption in attenuation of the beams and was related to the large atomic number of cerium. The simultaneous presence of these compounds in the nanocomposite structure will activate both mechanisms at the same time, and as a result, the attenuation efficiency will be higher.

In each category of non-hybrid nanocomposite films, with an increasing percentage of nanoparticles, the attenuation rate improved, which proved the effect of nanoparticles in attenuating the beams. On the other hand, excessive nanoparticles could affect the mechanical properties of the films by creating defects in the structure of the nanocomposite (These defects can be seen in some FESEM images such as M_7_ and M_10_). Therefore, increasing the percentage of nanoparticles in nanocomposites was limited.

Increasing the thickness of the layers can also have a significant effect on improving the attenuation performance of the films, so that quadrupling the thickness of the layers improves the attenuation efficiency by 60 to 77%.

Moreover, it was observed that the attenuation rate in hybrid films (despite the lower percentage of nanoparticles) was significantly higher than non-hybrid nanocomposite films and the synergistic effect of hybrid nanoparticles in radiation attenuation was well shown. This attenuation amplification may be related to overlapping between the Compton phenomenon and photoelectric absorption.

**Figure 9:**
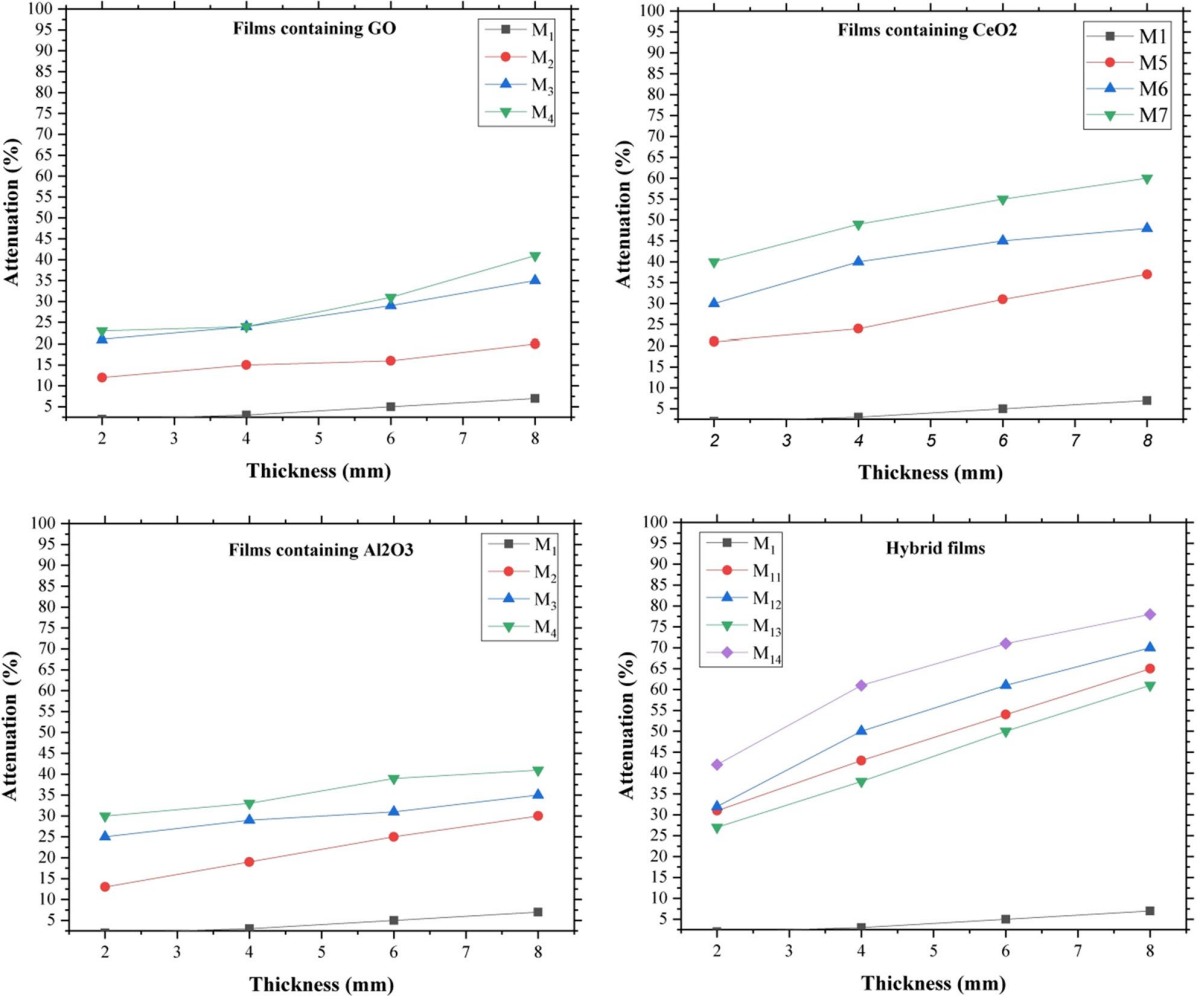
Attenuation of neat and hybrid nanocomposite films in terms of layer thickness in the broad beam geometry.

The results of film attenuation in terms of layer thickness in the broad beam geometry are shown in Fig. 9. The findings were quite similar to the results of narrow beam geometry, except that the attenuation rate in broad beam geometry was slightly lower, due to the possibility of detecting secondary scattered beams by the detector in this configuration.

**Figure 10:**
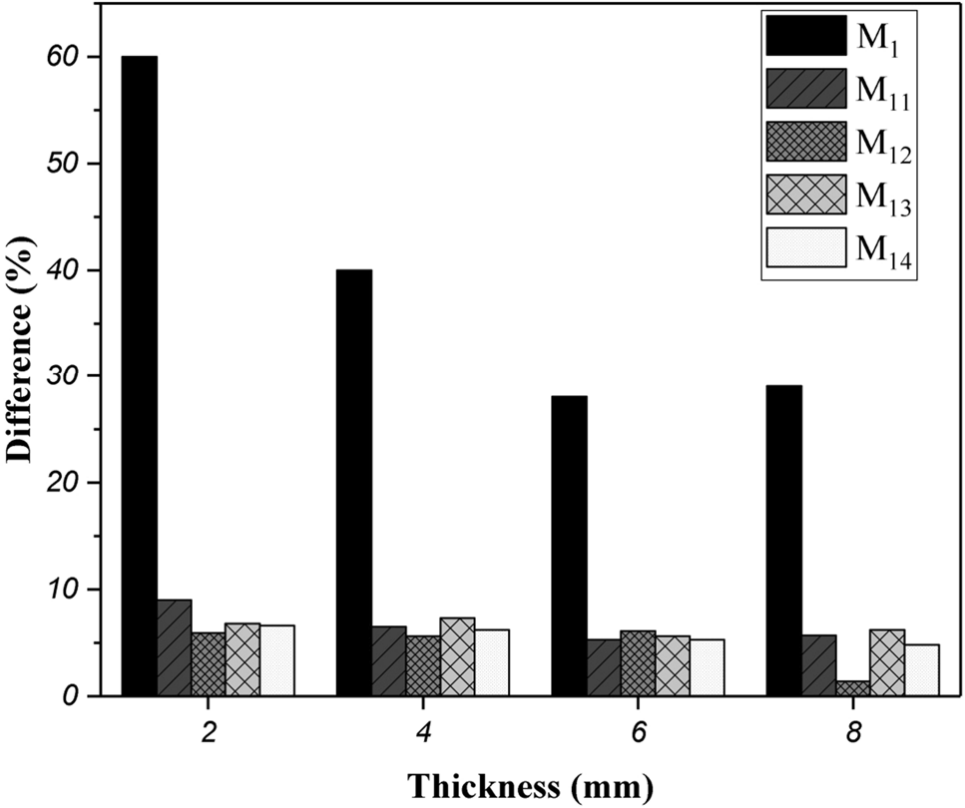
The attenuation difference between narrow and broad beam geometry for hybrid nanocomposite films.

The differences of attenuation in the two geometries for hybrid nanocomposite films are shown in Fig. 10 and compared with EVA neat film. This difference was more significant in pure polymer films, while in hybrid films, there was a much smaller difference in attenuation of the two geometries. This reduction is another evidence of the efficiency of nanoparticles in attenuating beams through two mechanisms and thus reducing the scattering of secondary beams.

**Figure 11:**
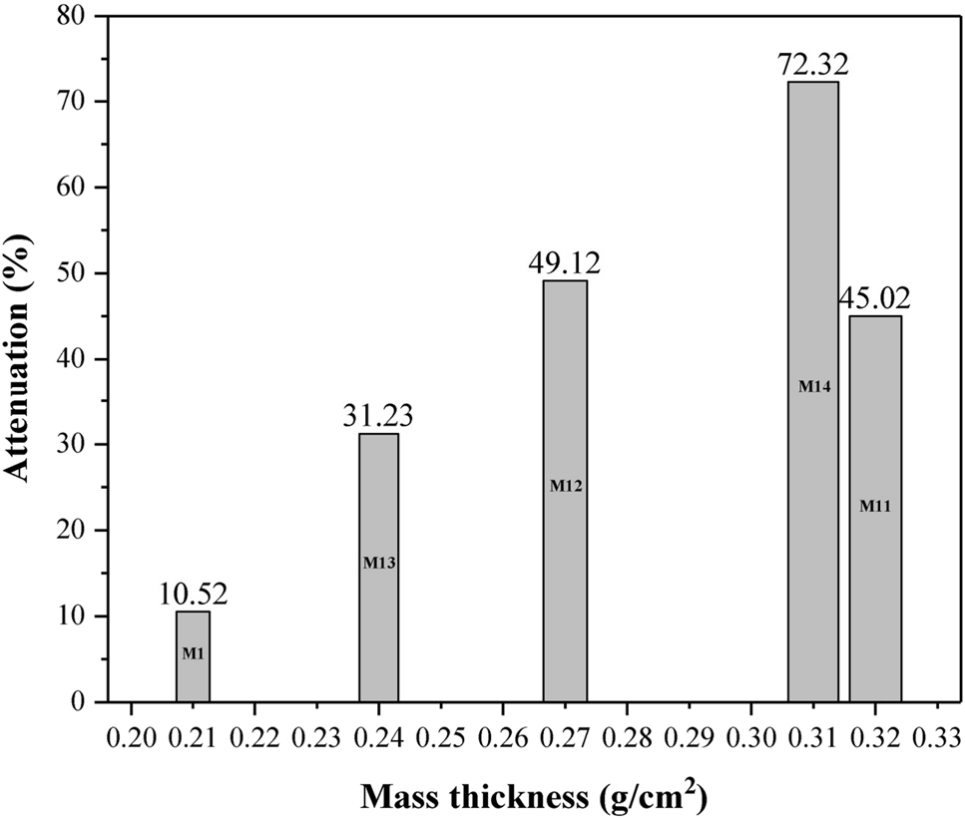
Attenuation in terms of mass thickness in pure and hybrid nanocomposite films.

Using the mass thickness coefficient (density multiplied by thickness (g/cm^2^)) in the Beer-Lambert equation, the attenuation will be calculated in terms of mass thickness. By entering the mass thickness coefficient, the effect of material density on attenuation is considered, and their attenuation capability is comparable regardless of density. In other words, it can be said that the mass is normalized to the surface area^40^. The attenuation of pure film and hybrid films in terms of mass thickness is shown in Fig. 11. As can be seen, films containing ceria nanoparticles with a higher mass thickness also had better attenuation efficiency. It should be noted that samples containing GO had a lower mass thickness than other hybrid films, and hybrid film M_14_ showed the highest removal efficiency despite having a lower mass thickness.

To ensure the accuracy of the results, standard pure lead samples with thicknesses of 0.25 and 0.5 mm were used to calculate the attenuation in narrow and wide beam geometries, and the results are given in Table 2. These results were compared with the data in other studies, and the consistency of the results confirmed the reliability of the calculated attenuations.

**Table 2:** Comparison of attenuation results of pure lead films with other studies.

Thickness (mm)	Attenuation (%)		
	This work	^41^	^11^
0.25	81.34	–	79.84
0.5	95.12	95.2	93.69

The attenuation rate of the optimal hybrid film M_14_ in this study was lower compared to the attenuation of the lead layer, while if the attenuation rate is calculated in terms of mass thickness, the attenuation obtained in this study will be comparable to the attenuation of the lead layer.

## Conclusion

In this study, EVA hybrid nanocomposite films were synthesized in the presence of ceria, GO, and α-alumina nanoparticles. Nanoparticles and fabricated films were characterized and studied by some analysis such as FTIR, FESEM, EDAX, and TGA. Examination of the morphology of the films showed that with increasing the percentage of nanoparticles in non-hybrid films, the accumulations between the nanoparticles caused defects in the structure of the nanocomposite. The attenuation ability of films against high-energy X-rays was evaluated in two configurations of narrow and broad beam geometry and it was observed that the attenuation rate improved with increasing the percentage of nanoparticles. Hybrid films, despite having lower percentages of nanoparticles, showed better attenuation efficiency, which was attributed to their synergistic effect in radiation attenuation due to the two mechanisms of photoelectric absorption, and the Compton phenomenon. The study of attenuation in terms of mass thickness indicated that films containing GO with lower mass thickness have better efficiency.

### Supplementary Information

Supplementary Figures.

## Data Availability

The datasets used and/or analysed during the current study available from the corresponding author on reasonable request.
