# Supplementary material for: Attenuation properties of hybrid nanocomposite film containing Ce2O, GO, and α-Al2O3 nanoparticles for high energy radiations
Source: Sci Rep. 2023 Sep 23;13:15918. doi: 10.1038/s41598-023-43212-9 (PMC10517928; doi:10.1038/s41598-023-43212-9)
Supplement: Supplementary file 1 — Supplementary Figures. [file 41598_2023_43212_MOESM1_ESM.pdf]

Attenuation Properties of Hybrid Nanocomposite Film Containing Ce_2O, GO, and α-Al_2O_3 Nanoparticles for High Energy Radiations

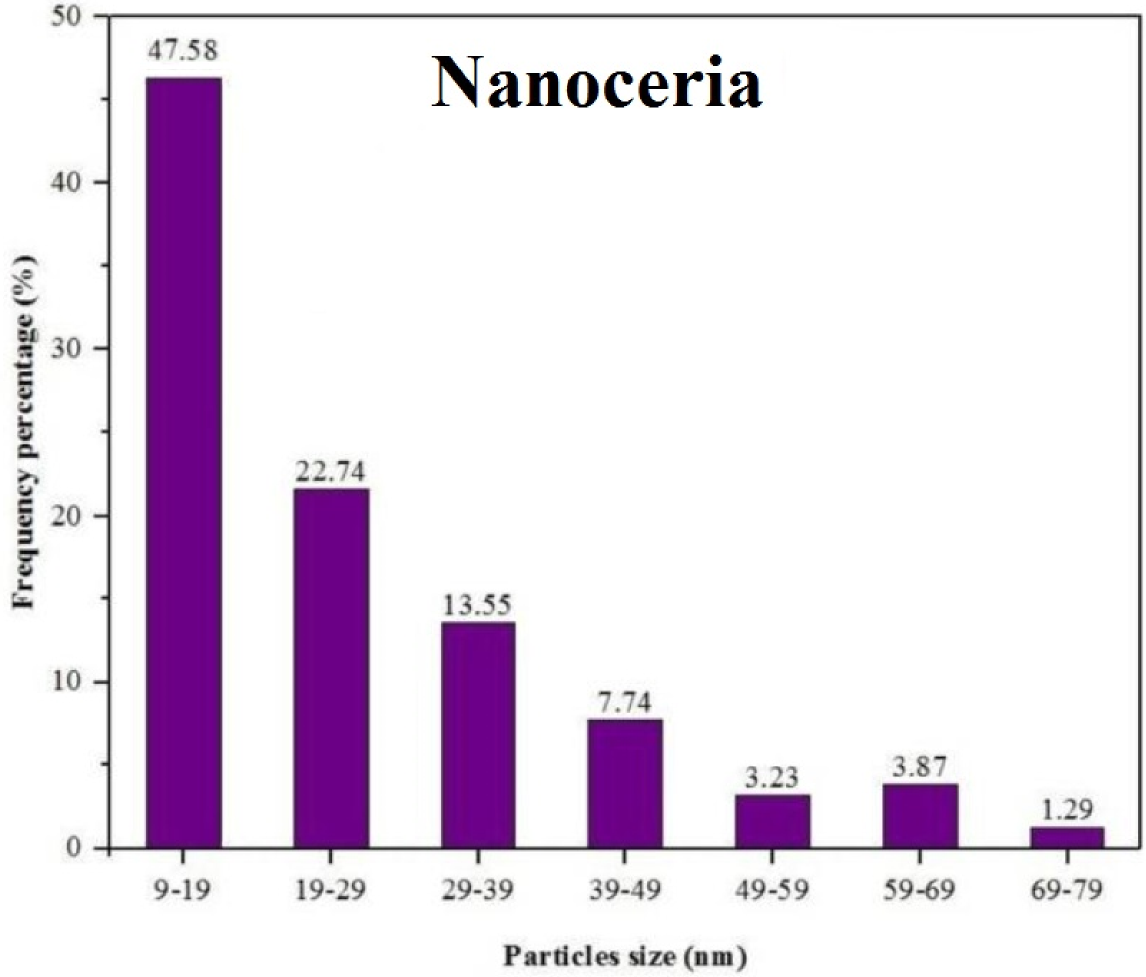

Figure S1. Particle size distribution for Nanoceria

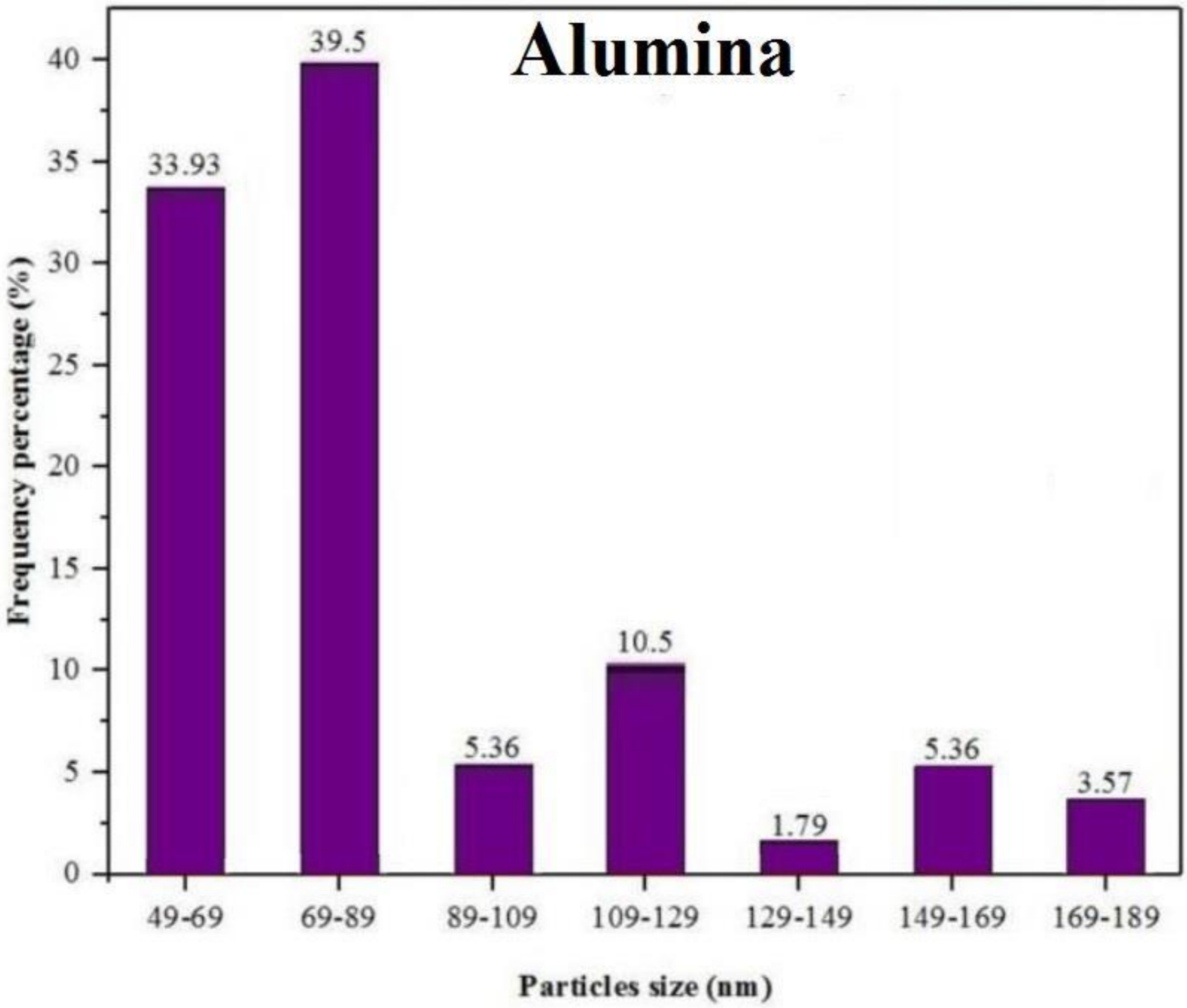

Figure S2. Particle size distribution for Alumina
